# Lycopene Protects Intestinal Epithelium from Deoxynivalenol-Induced Oxidative Damage via Regulating Keap1/Nrf2 Signaling

**DOI:** 10.3390/antiox10091493

**Published:** 2021-09-18

**Authors:** Shahid Ali Rajput, Shao-Jie Liang, Xiu-Qi Wang, Hui-Chao Yan

**Affiliations:** Department of Animal Nutrition and Feed Sciences, College of Animal Science, South China Agricultural University/Guangdong Laboratory for Lingnan Modern Agriculture/Guangdong Provincial Key Laboratory of Animal Nutrition Control/National Engineering Research Center for Breeding Swine Industry, Guangzhou 540642, China; dr.shahidali@scau.edu.cn (S.A.R.); liangsj@stu.scau.edu.cn (S.-J.L.)

**Keywords:** deoxynivalenol, lycopene, bioactive compound, intestinal injury, oxidative stress, Keap1/Nrf2 signaling

## Abstract

Deoxynivalenol (DON) is a threatening mycotoxin primarily present in the agricultural environment, especially in food commodities and animal forages, and exerts significant global health hazards. Lycopene (LYC) is a potent antioxidant carotenoid mainly present in tomatoes and other fruits with enormous health benefits. The present study was designed to ascertain whether LYC could protect DON-induced intestinal epithelium oxidative injury by regulating Keap1/Nrf2 signaling in the intestine of mice. A total of forty-eight mice were randomly distributed into four groups (*n* = 12), Control (CON), 10 mg/kg BW LYC, 3 mg/kg BW DON, and 3 mg/kg DON + 10 mg/kg LYC BW (DON + LYC). The experimental groups were treated by intragastric administration for 11 days. Our results showed that LYC significantly increased average daily feed intake (ADFI), average daily gain (ADG), and repaired intestinal injury and barrier dysfunction, as evident by increased trans-epithelial electrical resistance (TEER) and decreased diamine oxidase (DAO) activity, as well as up-regulated tight junction proteins (occludin, claudin-1) under DON exposure. Furthermore, LYC treatment stabilized the functions of intestinal epithelial cells (Lgr5, PCNA, MUC2, LYZ, and Villin) under DON exposure. Additionally, LYC alleviated DON-induced oxidative stress by reducing ROS and MDA accumulation and enhancing the activity of antioxidant enzymes (CAT, T-SOD, T-AOC, and GSH-Px), which was linked with the activation of Nrf2 signaling and degradation of Keap1 expression. Conclusively, our findings demonstrated that LYC protects intestinal epithelium from oxidative injury by modulating the Keap1/Nrf2 signaling pathway under DON exposure. These novel findings could lead to future research into the therapeutic use of LYC to protect the DON-induced harmful effects in humans and/or animals.

## 1. Introduction

Food safety is a global challenge to fulfill dire human needs. The research impetus is focused on the food contamination caused by the mycotoxins commonly present in the global environment [1,2]. Mycotoxins pose significant harm to human and animal health since they are major hazardous factors contaminating 25% of global crop production [3]. Deoxynivalenol (DON, vomitoxin) is a trichothecenes mycotoxin, primarily produced by *Fusarium graminearum* and *Fusarium culmorum* [4,5]. DON is frequently present in the agricultural environment, especially in cereals and animal forages, and causes health hazards to humans and animals [6,7]. Many studies described the deleterious effects of DON on human and animal health, such as food refusal, emesis, growth retardation, immune system, and gastrointestinal tract disorders [8,9].

Intestinal health is of paramount importance for the healthy growth of humans/and or animals, and it reflects the status of the intestinal physical barrier and immune function. The intestinal epithelium is the first layer of protection against gut infection [5]; it also regulates various nutrients absorption and represents the defensive barrier against enteric pathogens and their toxins [10,11], or other naturally occurring contaminants, for instance, DON [12]. Several studies have revealed that DON can alter the gut structure [13], malfunction the intestinal barrier function [2], and affect the absorption of nutrients [8,14], consequently compromising intestinal health. Moreover, the intestinal mucosal epithelium consists of four main cell types: absorptive enterocytes, goblet cells, Paneth cells, and enteroendocrine cells, which play a vital role in maintaining intestinal homeostasis [15,16]. Previous studies have stated that DON could alter the functions of intestinal epithelial cells [5]. Additionally, oxidative stress is a key risk factor of DON-induced toxicity in the intestinal epithelium [17]. The Kelch-like ECH-associated protein 1 (Keap1)/nuclear factor erythroid 2-related factor 2 (Nrf2) pathway is involved in cell survival and defense against extracellular or intracellular stress [18]. Previously, it was observed that DON-induced intestinal oxidative injury is linked with dysregulation of Nrf2 signaling [19].

Growing evidence has suggested that functional food components could alleviate the intestinal oxidative damage induced by DON [19]. Lycopene (LYC) is a naturally occurring bioactive compound mainly present in tomatoes, red-colored fruits, and vegetables [20]. LYC is a lipid-soluble pigment of carotenoid and is readily absorbed in the intestine by passive diffusion; enterocytes were identified as a potential site of LYC isomerization in-vivo [21]. LYC, a potent antioxidant, can effectively quench singlet oxygen and scavenge free radicals due to its distinctive unsaturated long-chain molecular structure [22,23]. Additionally, LYC exerts a broad range of biological activities, comprising anti-carcinogenic, anti-inflammatory, anti-cardiovascular diseases, neuroprotective, and detoxification abilities in several experimental and epidemiological studies [23,24,25]. Previous reports revealed that LYC relives AFB1-induced liver damage via increasing hepatic antioxidation and detoxification capacity through Nrf2 signaling activation [25]. However, the shielding effects and underlying mechanisms of LYC against DON-induced intestinal epithelium damage have not been reported yet. The present study hypothesized that LYC could protect DON-induced intestinal epithelium oxidative injury by regulating Keap1/Nrf2 signaling in the intestine of mice. To the best of our knowledge, this is the first study to highlight the protective role of LYC against DON-induced intestinal epithelium injury in mice.

## 2. Materials and Methods

### 2.1. Chemicals and Antibodies

Deoxynivalenol (DON, #D0156), N-acetylcysteine (#616-91-1), SB202190 (#S7067), nicotinamide (#98-92-0), ethylenediaminetetraacetic acid disodium salt (EDTA, #6381-92-6) and 4′,6-diamidino-2- phenylindole (DAPI, #28718-90-3) were procured from Sigma-Aldrich (St. Louis, MI, USA). Lycopene (LYC, #C10579425) and corn oil (#805618) were supplied by Macklin Biochemical Co., Ltd. (Shanghai, China). LYC was dissolved in corn oil, and DON was dissolved in PBS. Fetal bovine serum (FBS, #10099-141), B27 supplement (#12587010), N2 supplement (#17502048), and streptomycin/penicillin (#10378016) were purchased from Gibco (Waltham, MA, USA). Matrigel (#354230) was acquired from BD Biosciences (San Jose, CA, USA). Y27632 (#04-0012) was obtained from Stemgent (Cambridge, MA, USA). Recombinant murine EGF (#53003-018) was procured from PeproTech (Rocky Hill, NJ, USA). CHIR99021(#S1263) and LY2157299 (#S2230) were obtained from (SelleckChem, Houston, TX). Beyo ECL Plus, a chemiluminescence detection kit, was obtained from Beyotime Institute of Biotechnology (Shanghai, China). The kits for DAO ((#A088-1), ROS (#E004), MDA (#A003-1), T-AOC (#A015), GSH-Px (#A005), CAT (#A007-1) and T-SOD (#A001) were obtained from (Jiancheng Bioengineering Institute, Nanjing China). Bicinchoninic acid assay kit was supplied by Thermo Fisher Scientific (Waltham, MA, USA).

The primary antibodies, including occludin (#331500) and claudin-1 (#374900), were obtained from Thermo Fisher Scientific (Waltham, MA, USA). Villin (sc-58897) and MUC2 (sc-15334) were procured from Santa Cruz Biotechnology (Santa Cruz, CA, USA). LYZ (A0099) was obtained from Dako (Copenhagen, Denmark). Lgr5 (TA503316) was acquired from OriGene Technologies (Rockwell, IA, USA). The Keap1 (#8047), HO-1 (#5853), NQO1 (#3187), and secondary antibodies used in the present study, including anti-mouse IgG (#4410) and anti-rabbit IgG (#4414), were procured from Cell Signaling Technology (Beverly, MA, USA). Nrf2 (#380773), p-Nrf2 (#381559), PCNA (#200947), and β-actin (#600149) were acquired from Zen BioScience (Chengdu, Sichuan, China). Cy3-conjugated (#111-165-045) and FITC (#115-545-003) antibodies were procured from Jackson Laboratory (Jackson, MS, USA).

### 2.2. Ethics Statement

All experimental procedures were approved by the Laboratory Animals Care and Use Committee of South China Agricultural University, Guangzhou, China (SCAU) (Protocol code # SCAU-0206). All the methods were carried out following the approved guidelines of SCAU.

### 2.3. Animals and Experimental Design

Healthy C57BL/6 mice were procured from Medical Experimental Animal Center (Foshan, China). Following a week of acclimatization period, a total of forty-eight mice were allocated randomly into four treatment groups (*n* = 12), as summarized in Table 1. All groups were treated with gavage administration once a day.

### 2.4. Sample Collection

After euthanizing, blood samples were harvested by retro-orbital puncture, separated by centrifugation for serum samples, and stored at −80 °C for a subsequent test. The intestine samples were excised immediately and washed with ice-cold PBS. Subsequently, the intestine samples were weighed and fixed in 2.5% glutaraldehyde or 4% fresh paraformaldehyde for morphological observation or immersed in liquid nitrogen and then preserved at −80 °C for further assessment. The rest of the jejunum tissue was used to isolate the crypts. LYC or DON dosages chosen in the current research were based on our preliminary experiment. Body gain, water intake, and feed intake were monitored throughout the experiment. Animals were kept under laboratory conditions at 22 ± 2 °C and were subjected to a controlled photoperiod (12 h light–12 h dark) and relative humidity of 45–60%. Pellet diet and water were provided ad libitum. Animal health was closely observed, and there were no signs of morbidity or mortality in any experimental mice.

### 2.5. Intestinal Crypt Isolation and Culture

The intestinal crypt isolation was performed based on our previous study [5]. In brief, jejunum samples were washed with DPBS and cut into about 5 cm segments. After that, they were incubated with DPBS containing 30 Mm ethylenediaminetetraacetic acid disodium salt (ETDA). The crypts were then transferred into 50 mL tubes, and fresh DPBS was added, and the process was repeated until high purity crypts were obtained. Finally, the purified crypts were cultured as previously described in our study [26]. The enteroids forming and budding efficiency were calculated by using Image-Pro Plus software.

### 2.6. Transepithelial Electrical Resistance (TEER) Assay

The TEER was determined by using an ECOM2 epithelial Volt/Ohm meter (Millipore, Billerica, MA, USA). Fresh jejunal samples were dissected into small fragments and balanced in Krebs–Ringer buffer. The jejunum segments were mounted between the two halves of a chamber and filled with an appropriate volume of Krebs–Ringer buffer on both sides. The system was constantly gassed with carbogen to maintain the tissue viability, and the temperature was sustained at 37 °C with a water jacket. Following a 30-min equilibration period, the solutions were changed with a new Krebs–Ringer buffer, and then the tests were performed. The data were expressed as Ω (resistance) × cm^2^ (surface area of the monolayer) after deducting the filter resistance value.

### 2.7. Hematoxylin and Eosin (H&E) Staining

The H&E staining was carried out as previously described [27]. Briefly, jejunum tissues were fixed in 4% paraformaldehyde, dehydrated with alcohol, and embedding was performed. The 4 µm fragments were sectioned and prepared for H&E staining. The morphometry of intestinal villus and crypt were measured using Image-Pro Plus software [26].

### 2.8. Scanning Electron Microscope (SEM)

SEM was performed following our previously reported procedure [28]. The jejunum tissues were soaked in 2.5% glutaraldehyde for 24 h, then washed in PBS and incubated with 1% osmium tetroxide in sodium cacodylate buffer for an hour. Next, the samples were dehydrated with an alcohol solvent and finally dried to the critical point. Afterward, the jejunum was pasted to stubs by carbon tape and covered with gold. The jejunum images were taken using an EVO MA 15 scanning electron microscope (Carl Zeiss AG, Jena, Germany).

### 2.9. Measurement of Oxidative Stress Indices and Diamine Oxidase Activity

For the oxidative stress indices, jejunum tissue homogenates were prepared following the corresponding kit requirements. BCA kit was used to measure the protein content of samples. Reactive oxygen species (ROS), Malondialdehyde (MDA), catalase (CAT), total superoxide dismutase (T-SOD), glutathione peroxidase (GSH-Px), total antioxidant capacity (T-AOC), and diamine oxidase (DAO) activity were detected using commercially available kits supplied by (Jiancheng Bioengineering Institute, Nanjing, China).

### 2.10. Immunohistochemistry (IHC) Analysis

The immunohistochemical analysis was performed as described by [28]. First, the stained sections were incubated with primary antibodies at 4 °C for overnight. Later, the sections were washed three times in PBS, each for 5 min. After washing, the sections were incubated with secondary antibodies for two h at room temperature, then washed with PBS thrice. Finally, DAPI was used for staining the nuclei for 10 min at room temperature. The images were observed using a fluorescence microscope (Nikon, Tokyo, Japan). Quantification was performed by Image-Pro plus software.

### 2.11. Western Blotting

Western blotting was conducted as we described previously [29]. In short, SDS-PAGE was used to separate the jejunum and crypt proteins. Then, the samples were shifted to PVDF membranes and blocked with 5% skim milk; subsequently, the membranes were incubated overnight at 4 °C with primary antibodies. Finally, following three washes with TBST, membranes were incubated with secondary antibodies for visualization [30].

### 2.12. Automated Capillary Western Blotting (WES)

WES was conducted in accordance with our earlier described procedure [31]. Enteroids samples were lysed with RIPA buffer and mixed with a 5 × fluorescent master mix and then warmed for 5 min at 95 °C. Finally, the diluted protein lysate sample, washing buffer, blocking reagent, primary and secondary antibodies, and chemiluminescent substrate are distributed to the specified wells. The default settings were used for automatic protein separation in each capillary. The data were examined by using Compass software 3.1 (ProteinSimple, San Jose, CA, USA).

### 2.13. Statistical Analysis

The data were analyzed using SPSS (version 22, IBM Corporation, Armonk, NY, USA) software. Statistical analyses were performed using one-way ANOVA followed by the least significant difference (LSD) test. The results are presented as mean ± SEM. The differences among groups were considered statistically significant at *p*-value < 0.05.

## 3. Results

### 3.1. LYC Treatment Improves Growth Performance of Mice Exposed to DON

**Figure 1:**
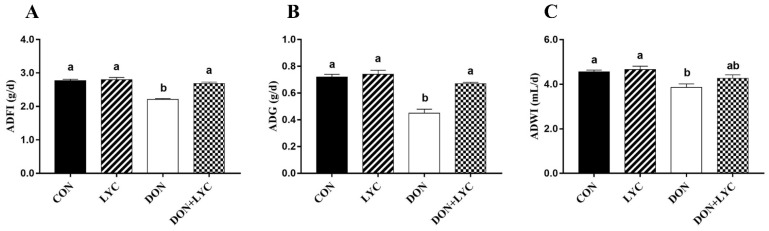
LYC treatment improves the growth performance of mice exposed to DON. (**A**) ADF1, (**B**) ADG, and (**C**) ADWI. The results are presented as mean ± SEM (*n* = 12). Columns with different superscripts letters indicating significant difference (*p* < 0.05).

To evaluate the beneficial effect of LYC treatment on DON-induced growth retardation in mice, the ADFI, ADG, and ADWI were monitored during the experiment. The ADFI, ADG, and ADWI in the DON group were significantly lower than those of the CON group (*p* < 0.05). On the other hand, compared to the DON challenged group, LYC treatment increased the ADFI (*p* < 0.05) (Figure 1A) and ADG (*p* < 0.05) (Figure 1B), while no significant difference was found on the ADWI of mice. These findings demonstrated that LYC treatment could eliminate the toxic effects of DON on mice growth performance.

### 3.2. LYC Treatment Repairs Intestinal Epithelium Injury of Mice Exposed to DON

**Figure 2:**
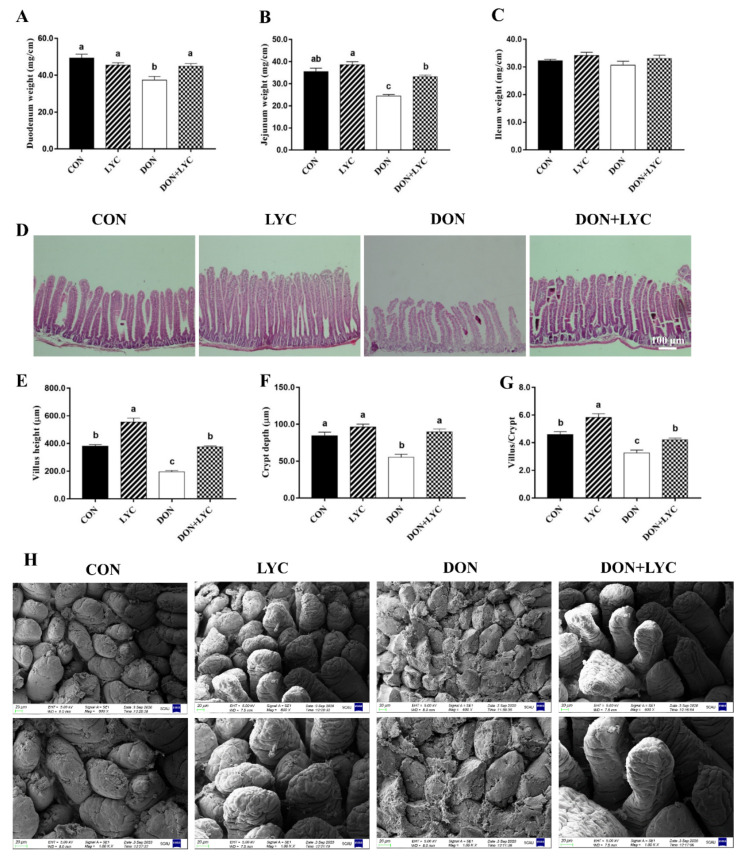
Lycopene treatment repairs intestinal epithelium injury of mice exposed to DON. (**A**) Duodenum weight (**B**), jejunum weight, and (**C**) ileum weight. Results are expressed as mean ± SEM (*n* = 6). (**D**) Hematoxylin and eosin (H&E) staining in the jejunum. (**E**) Villus height, (**F**) crypt depth, and (**G**) ratio of the villus to the crypt. The results are presented as mean ± SEM (*n* = 3). (**H**) Represented images of scanning electron microscopy in the jejunum of mice (600× and 1000×). Columns with different superscripts letters indicating significant difference (*p* < 0.05).

To explore the shielding role of LYC on DON-induced intestinal epithelial injury of mice, we investigated the growth or wound healing of the intestinal epithelium. As shown in Figure 2A–C, DON decreased the duodenum and jejunum weight significantly without affecting the ileum weight compared to the CON group. On the other hand, LYC considerably increased the duodenum (*p* < 0.05) and jejunum (*p* < 0.05) weight of mice as compared to the DON group. The morphological changes in the jejunum of mice are depicted (Figure 2D,H). The morphological results revealed that DON challenged group displayed severe atrophy, cell exfoliation, and multifocal apical necrosis of the villi in the jejunum of mice. Interestingly, LYC treatment significantly reversed the DON-induced morphological alterations, as presented in the ordered crypt-villus axis architecture in the jejunum of mice. Conversely, the LYC treatment markedly increased the villus height (*p* < 0.05) and crypt depth (*p* < 0.05), as well as villus/crypt ratio (*p* < 0.05) in the jejunum of mice decreased by DON (Figure 2E–G).

### 3.3. LYC Treatment Protects the Intestinal Barrier Disruption of Mice Exposed to DON

**Figure 3:**
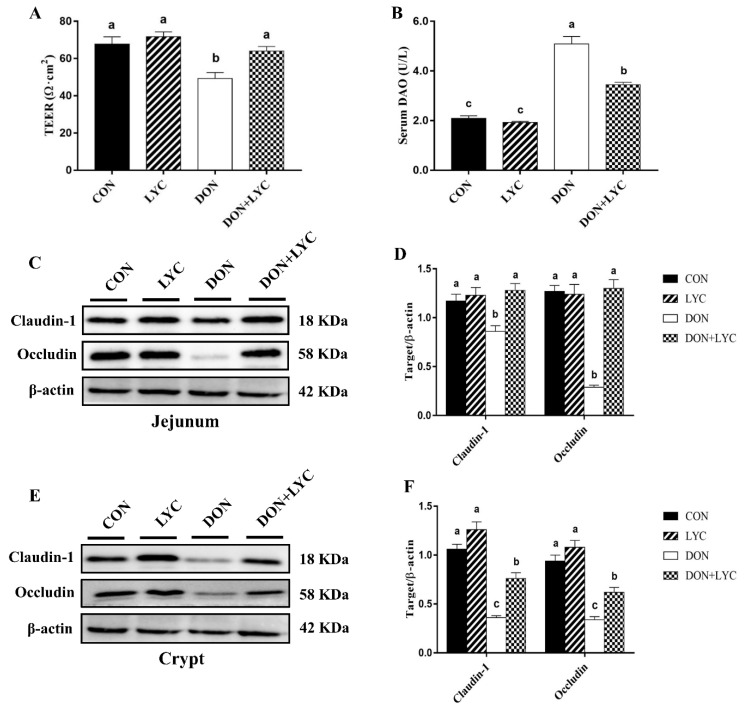
LYC treatment prevents the intestinal barrier disruption of mice induced by DON. (**A**) TEER in the jejunum. Results are presented as mean ± SEM (*n* = 3). (**B**) DAO activity in the serum. The results are expressed as mean ± SEM (*n* = 6). (**C**,**D**) Protein expression of claudin-1 and occludin in the jejunum and (**E**,**F**) protein expression of claudin-1 and occludin in the crypt. Results are presented as mean ± SEM (*n* = 3). Columns with different superscripts letters indicating significant difference (*p* < 0.05). TEER, Trans-epithelial electrical resistance; DAO, diamine oxidase.

To identify the protective effects of LYC on DON-induced gut barrier dysfunction of mice, we examined the trans-epithelial electrical resistance (TEER) in the jejunal tissue and diamine oxidase (DAO) activity in the serum of mice. Our results reflected that DON exposure significantly declined TEER in the jejunum (*p* < 0.05) (Figure 3A), while DAO activity was (*p* < 0.05) increased in the serum (Figure 3B) in the comparison of CON group, while LYC treatment considerably reversed these changes altered by DON (*p* < 0.05).

To further investigate the LYC-mediated defensive effects on DON-induced gut barrier dysfunction are implicated with tight junction proteins, the claudin-1 and occludin protein expression in the jejunal tissue and crypt of mice were measured by Western blotting. The LYC treatment significantly up-regulated the claudin-1 and occludin protein expression in the jejunum (*p* < 0.05) (Figure 3C,D) and crypt (*p* < 0.05) (Figure 3E,F) down-regulated by DON. These findings indicated that LYC mitigated the intestinal barrier disruption induced by DON.

### 3.4. LYC Treatment Improves the Growth Advantages of Enteroids under DON Exposure

**Figure 4:**
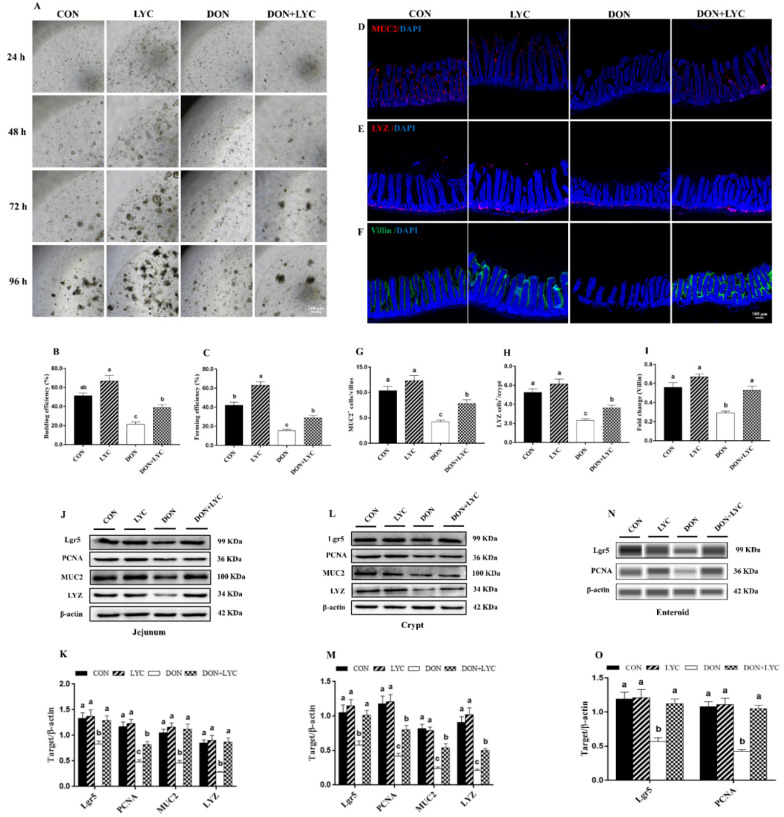
LYC treatment stabilized the functions of intestinal epithelial cells under DON exposure. (**A**) Represented images of enteroids expended from crypt stem cells. (**B**,**C**) Enteroid forming and budding efficiency. (**D**–**F**) Represented images of immunohistochemistry staining with MUC2, LYZ, and Villin antibodies in the jejunum. (**G**,**H**) Statistical analysis of MUC2+ cells and LYZ+ cells. (**I**) Statistical analysis of fluorescence intensity of Villin. (**J**,**K**) Protein expression of PCNA, Lgr5, MUC2, and LYZ in the jejunum. (**L**,**M**) Protein expression of PCNA, Lgr5, MUC2, and LYZ in the crypt. (**N**,**O**) Protein expression of Lgr5 and PCNA in the enteroids of mice. Results are presented as mean ± SEM (*n* = 3). Columns with different superscripts letters indicating significant difference (*p* < 0.05).

The jejunal crypts from the CON and experimental groups of mice were isolated and cultured. As shown in Figure 4A–C, DON exposure significantly declined the enteroids forming and budding efficiency (*p* < 0.05) compared to the CON group. However, LYC significantly (*p* < 0.05) improved the enteroids expansion reduced by DON. Furthermore, our results revealed that LYC treatment significantly up-regulated Lgr5 expression (active ISC marker) in the jejunum (*p* < 0.05) (Figure 4J,K), crypt (*p* < 0.05) (Figure 4L,M), and enteroids (*p* < 0.05) (Figure 4N,O) of mice in the comparison of DON group. The above findings suggested that LYC protects intestinal epithelium integrity by maintaining ISC activity under DON exposure.

### 3.5. LYC Treatment Stabilized the State of Intestinal Epithelial Functional Cells under DON Exposure

Proliferative and differentiative cell markers are the essential components for maintaining intestinal epithelium survival. To highlight the effect of LYC on intestinal epithelial cell proliferation and differentiation, the protein expression and fluorescence intensity of PCNA, MUC2, LYZ, and Villin were detected. In comparison to the DON group, LYC dramatically up-regulated the MUC2, LYZ, and PCNA protein expression in the jejunum, crypt, and enteroids of mice (*p* < 0.05) (Figure 4J–O). We further detected the number of MUC2+ and LYZ+ cells and the fluorescent intensity of Villin. DON exposure (*p* < 0.05) decreased MUC2+ cells in the villi and LYZ+ cells in the crypt. Additionally, the fluorescence intensity of Villin was also declined by DON (*p* < 0.05). On the other hand, LYC treatment significantly reversed the DON-induced changes in the MUC2+ cells, LYZ+ cells, and Villin fluorescence intensity in the jejunum of mice (Figure 4D–I).

### 3.6. LYC Treatment Attenuated Intestinal Epithelium Oxidative Damage Induced by DON

**Figure 5:**
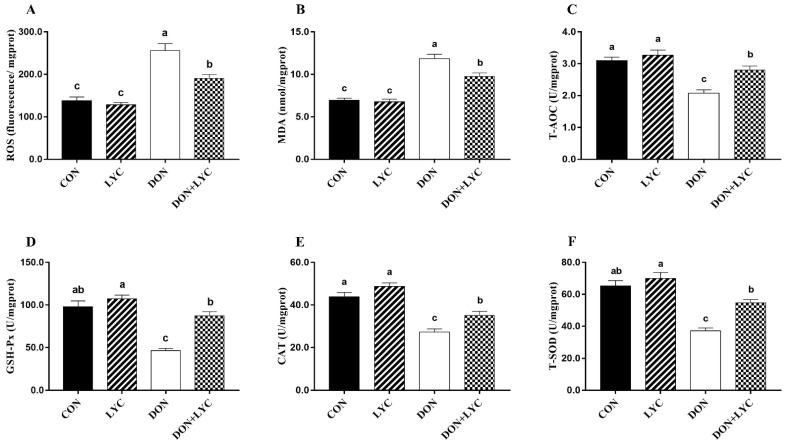
LYC treatment attenuated intestinal epithelium oxidative injury induced by DON. (**A**) Reactive oxygen species (ROS), (**B**) malondialdehyde (MDA), (**C**) total antioxidant capacity (T-AOC), (**D**) glutathione peroxidase (GSH-Px), (**E**) catalase (CAT), and (**F**) total superoxidase dismutase (T-SOD). Results are presented as mean ± SEM (*n* = 6). Columns with different superscripts letters indicating significant difference (*p* < 0.05).

To detect the redox status in the intestinal epithelium of experimental mice, the levels of ROS and MDA, as well as the CAT, T-SOD, T-AOC, and GSH-Px were measured. As shown in Figure 5, in the comparison of CON group, ROS and MDA levels were raised in response to DON exposure (*p* < 0.05). However, LYC treatment significantly (*p* < 0.05) mitigated ROS and MDA levels in the jejunum of mice compared with the DON treated group. Moreover, DON exposure markedly declined the CAT, T-SOD, T-AOC, and GSH-Px activities (*p* < 0.05) compared with the CON group. In contrast, LYC treatment revealed a significant increase in CAT, T-SOD, T-AOC, and GSH-Px, compared to the DON group (Figure 5A–F).

### 3.7. LYC Treatment Promoted DON-Induced Nrf2 Signaling Activation via Down-Regulation of Keap1

**Figure 6:**
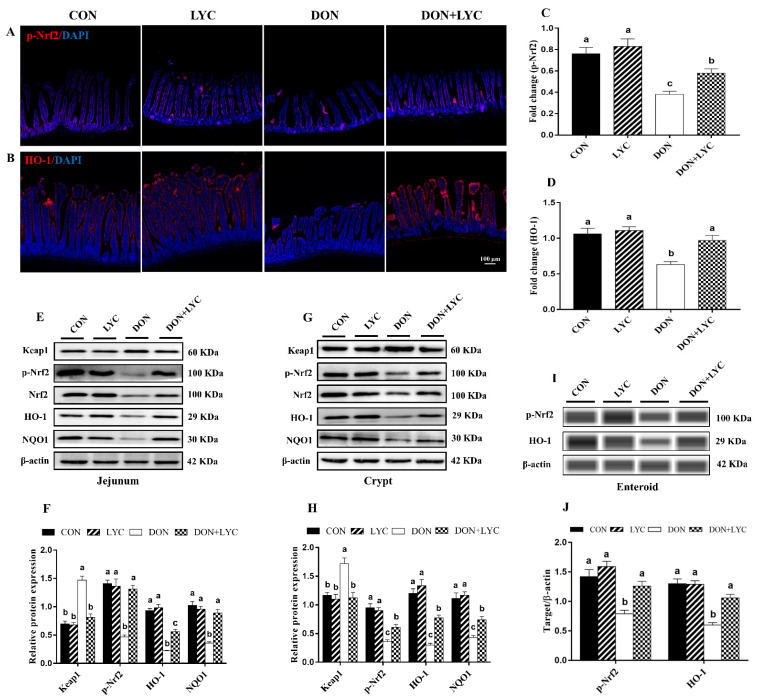
LYC treatment promoted DON-induced Nrf2 signaling activation via down-regulation of Keap1. (**A**,**B**) Represented images of immunohistochemistry staining with p-Nrf2 and HO-1 antibodies in the jejunum of mice. (**C**,**D**) Statistical analysis of the fluorescence intensity of p-Nrf2 and HO-1. (**E**,**F**) Protein expression of Keap1, p-Nrf2, Nrf2, HO-1, and NQO1 in the jejunum. (**G**,**H**) Protein expression of Keap1, p-Nrf2, Nrf2, HO-1, and NQO1 in the crypt. (**I**,**J**) Protein expression of p-Nrf2 and HO-1 in the enteroids of mice. All the data are expressed as mean ± SEM (*n* = 3). Columns with different superscripts letters indicating significant difference (*p* < 0.05).

To confirm our hypothesis of whether LYC maintains the redox homeostasis of intestinal epithelial cells against DON-induced oxidative stress is linked with Nrf2 activation, the protein expression of Keap1, p-Nrf2, HO-1 and NQO1 were detected. Our findings showed that DON treatment (*p* < 0.05) increased the Keap1 protein expression; however, this effect was reversed considerably with the treatment of LYC (*p* < 0.05) (Figure 6E,H). In the DON group, the protein expression and fluorescence intensity of p-Nrf2, HO-1, and NQO1 were down-regulated (*p* < 0.05) in comparison to the CON group. In contrast to the DON treated group, LYC markedly (*p* < 0.05) up-regulates the fluorescence intensity (Figure 6A,D) and expression of p-Nrf2, HO-1, and NQO1 in the jejunum (Figure 6E,F), crypt (Figure 6G,H) and enteroids (Figure 6I,J) of mice.

## 4. Discussion

The DON is recognized to contaminate a diversity of food and feedstuffs, posing serious hazards to the agricultural environment and public health [32]. DON is rapidly absorbed in the small intestine in the majority of animals by passive diffusion, particularly proximal jejunum, so the intestinal epithelium is the primary target of DON [33]. Therefore, it is imperative to find an effective protective agent to shield the intestinal epithelium from DON-induced toxicity. The LYC, well known as a phytochemical agent, is primarily present in vegetables and fruits with enormous health benefits [34]. After ingestion, LYC is isomerized into Cis-configuration in the GIT and finally absorbed in the gut via intestinal epithelial cells [35]. We found that DON exposure caused poor growth performance of mice. These results may be attributed to the DON-induced intestinal epithelium injury. Intestinal morphology is considered one of the most key parameters to reveal intestinal health and injury [13,36]. The villus height and crypt depth indicate the digestive and absorption capacity of the intestinal epithelial cells [26]. In our study, DON exposure decreased the duodenum and jejunum weight, as well as declined the villus height, crypt depth, and villus to crypt ratio in the jejunum of mice. Our findings indicated that DON could cause intestinal injury and suppress the growth performance of mice. These results can be explained, as DON exposure could shorten the villi, resulting in poor nutrient absorption, diarrhea and consequently lead to poor growth performance [37]. Previously, LYC supplementation prevented methotrexate-induced intestinal injury by maintaining intestinal structure in rats [38]. Our results showed that LYC treatment significantly repaired intestinal injury by increasing villus height and crypt depth resulted in better nutrient absorption, suggesting that LYC alleviated DON-induced toxicity on the growth performance of mice.

The integrity of the epithelial cells layer is essential for gut barrier function. Recently, LYC has appeared to be an essential functional nutrient for intestinal integrity [39,40]. In the present research, TEER in the jejunum was significantly reduced, while DAO content in the serum markedly increased after DON exposure. Our results indicated that DON exposure debilitates gut barrier function and concedes an invasion of exogenous noxious agents present in food or feed [12,41]. Additionally, the intestinal barrier function is closely linked to the junctional proteins network, and several studies have suggested that DON alters tight junction (TJ) proteins [2,26]. However, we observed that LYC treatment protects DON-induced gut barrier disruption by improving the structural integrity and promoting TJ proteins (occludin and claudin-1). Moreover, the intestinal epithelium is considered one of the fastest self-renewing tissue in the mammalian body. In our research, LYC significantly protects intestinal epithelium integrity by enhancing the growth advantages of enteroids under DON exposure. In addition, considerable evidence suggested that Goblet cells (MUC2) and Paneth cells (LYZ) act as a layer of protection for epithelial cells, also implicated in resistance to harmful endogenous agents and their toxins [5]. We found that LYC rescued MUC2, LYZ and PCNA expression and fluorescence intensity of Villin under DON exposure, suggesting LYC stabilized the functions of intestinal epithelial cells. Similarly, previously, LYC protected intestinal injury induced by sulfamethoxazole [35]. Another study demonstrated that LYC treatment prevented dextran sulfate sodium-induced gut barrier damages and inflammatory responses in male rats [40]. In the current research, the increased number of MUC2+ cells in the villi and LYZ+ cells in the crypt further indicate that the LYC can protect barrier function in the small intestinal mucosa.

Oxidative stress is typically caused by an imbalance between prooxidants and antioxidants, contributing to gut diseases, consequently, intestinal barrier dysfunction [19,42]. The overproduction of ROS or decreased antioxidant capacity usually caused oxidative stress, leading to severe cell injury and/or cell death in humans or animals [17]. Previously, it was reported that DON exposure augmented the ROS generation and inhibited the antioxidant enzyme activities (GSH-Px, T-SOD) in IPEC-J2 cells [33], consistent with our findings. Antioxidant enzymes play a critical role in the body’s antioxidant defense system by removing ROS from the cell [43,44]. LYC is a potent antioxidant that effectively scavenges ROS accumulation, protecting against cell damage caused by oxidative stress [23]. The present results demonstrated that LYC significantly reduced ROS and MDA accumulation while improving antioxidant enzyme activities (CAT, T-SOD, T-AOC, GSH-Px) under DON exposure.

Nrf2 is a transcription factor and plays a crucial role in enhancing cell protection against oxidative damage [25,45]. Normally, Nrf2 is sequestered in the cytoplasm by its specific negative regulator Keap1. However, under stimulation, Nrf2 dissociates from Keap1 and translocates to the nucleus, where it binds antioxidant response elements (ARE) and promotes its associated targets (NQO-1, HO-1, GCLC) [46]. Most notably, Nrf2 appears to play a central role in protecting intestinal epithelium integrity against some toxic agents [47]. Previously, Nrf2 was involved in DON-induced oxidative injury in the intestinal epithelium [19]. In this research, DON exposure significantly up-regulated the Keap1 expression and suppressed Nrf2 as well as its downstream targets (HO-1, NQO1). There might be some reasons why DON inhibited the nuclear translocation of Nrf2. It could be involved with the suppression of de novo synthesis of Nrf2 induced by DON. However, the specific mechanism remains to be clarified. We found that LYC treatment significantly reversed these effects caused by DON. The current investigation is in line with the earlier studies, stating that DON exposure suppressed Nrf2 nuclear translocation [19,48]. LYC could protect cells from oxidative damage by enhancing Nrf2 translocation into the nucleus, thereby increasing the cellular antioxidant gene expression [25,49,50]. The current study suggested that Nrf2 activation is one of the key mechanisms underlying the LYC beneficial effects. However, the mechanism by which LYC activates Nrf2 needs to be further researched.

## 5. Conclusions

**Figure 7:**
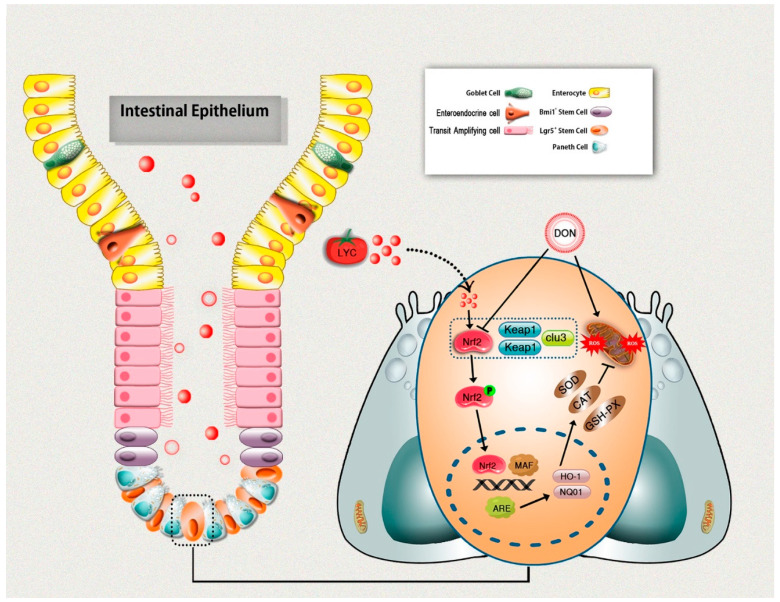
Schematic diagram representing lycopene-induced protection of intestinal epithelium integrity through regulation of Keap1/Nrf2 signaling pathway under DON exposure.

The current study provides significant evidence on the potential protective effects of LYC against DON-induced intestinal epithelium injuries in mice. LYC effectively repaired DON-induced intestinal damages, as evident from the improved intestinal structure and gut barrier functions. Moreover, LYC alleviated DON-induced intestinal epithelium oxidative injury by scavenging ROS production and enhancing the antioxidant defense system, which is probably linked with Keap1/Nrf2 signaling pathway regulation. In return, this protects intestinal epithelium injury induced by DON (Figure 7). These novel findings could lead to future research into the therapeutic use of LYC to protect the DON-induced hazardous effects in humans and/or animals.

## Figures and Tables

**Table 1:** Experimental design.

Treatment	Days											
	1	2	3	4	5	6	7	8	9	10	11	12
CON	OIL			OIL					OIL			Euthanized
LYC	LYC			LYC					LYC			Euthanized
DON	OIL			DON					OIL			Euthanized
DON+LYC	LYC			DON+LYC					LYC			Euthanized

CON (corn oil); LYC (10 mg/Kg BW); DON (3 mg/Kg BW); DON+LYC (DON 3 mg/Kg + LYC 10 mg/Kg BW).

## Data Availability

Data is contained within the article.
